# Consumption of foods with the Keyhole front-of-pack nutrition label: potential impact on energy and nutrient intakes of Swedish adolescents

**DOI:** 10.1017/S1368980022002178

**Published:** 2022-12

**Authors:** Julia Wanselius, Christel Larsson, Christina Berg, Veronica Öhrvik, Anna Karin Lindroos, Lauren Lissner

**Affiliations:** 1 Department of Food and Nutrition, and Sport Science, University of Gothenburg, Läroverksgatan 5 Box 300, SE-405 30, Gothenburg, Sweden; 2 Science Division, Swedish Food Agency, Uppsala, Sweden; 3 Axfoundation, Torsåker, Sweden; 4 Department of Internal Medicine and Clinical Nutrition, Institute of Medicine, Sahlgrenska Academy, University of Gothenburg, Gothenburg, Sweden; 5 School of Public Health and Community Medicine, Institute of Medicine, Sahlgrenska Academy, University of Gothenburg, Gothenburg, Sweden

**Keywords:** Nutrient profiling, Front-of-pack nutrition label, Food labelling, Nutritional information, Adolescents

## Abstract

**Objective:**

The Keyhole is an internationally recognised front-of-pack nutrition label, guiding consumers to healthier food options. It indicates products in accordance with specific criteria for dietary fats, sugars, fibres, salt and wholegrains. The objective of this study was to simulate the potential impact of the Keyhole on adolescents’ energy and nutrient intakes by modelling a shift from reported food intakes to foods meeting the Keyhole criteria.

**Design:**

Self-reported dietary intake data were derived from a cross-sectional survey. Multiple replacement scenarios were calculated, where foods meeting the Keyhole criteria replaced reported non-compliant foods with varying proportions of replacement.

**Setting:**

Dietary survey ‘Riksmaten Adolescents 2016–2017’ in schools across Sweden.

**Participants:**

A nationally representative sample of 3099 adolescents in school years 5, 8 and 11 (55 % girls).

**Results:**

Overall, replacement with foods meeting the Keyhole criteria led to more adolescents meeting nutrition recommendations. Largest median intake improvements were seen for wholegrains (+196 %), SFA (-13 %), PUFA (+17 %) and fibres (+15 %). Smallest improvements were seen for free sugars (-3 %) and salt (-2 %), partly explained by the ineligibility of main food sources of free sugars for the Keyhole, and non-inclusion of ready meals that are often high in salt. Most micronutrient intakes were stable or improved. Unintentional effects included decreases in vitamin A, MUFA and energy intakes. Largest potential improvements in fat and fibre sources were observed in the youngest age group.

**Conclusions:**

A shift to Keyhole alternatives for everyday foods would improve adolescents’ nutrient intakes, even with smaller exchanges.

Adolescents’ dietary intakes in Sweden are not in line with dietary guidelines. According to the latest national survey conducted by the Swedish Food Agency, insufficient intakes of vegetables and fruits have been observed^(1)^, together with excessive intakes of discretionary, high-energy, low-nutrient foods such as sweets, cookies, snacks and sugar-sweetened beverages^(2)^. The prevalence of overweight and obesity in adolescents has been rising internationally^(3)^ including in Sweden^(4)^ where one-fifth of adolescents are either overweight or obese^(1)^. On average, adolescents’ diets also include too much SFA, salt and free sugars, while being too low in PUFA, dietary fibre and wholegrains^(5)^.

One available tool for promoting healthy food habits is nutrient profiling, which is defined by the WHO as ‘the science of classifying or ranking foods according to their nutritional composition for reasons related to preventing disease and promoting health’^(6)^. Nutrient profiling is typically used either to describe nutrient levels in foods (e.g. ‘high fat’ and ‘low fat’) or refers to overall health properties such as being a more or less healthy option. Nutrient profiling is commonly applied to nutrition labelling, in which front-of-pack (FOP) or back-of-pack nutrition labels assist the consumer towards making healthier food choices^(7)^. These nutrition labels are a good complement to the nutrition facts panels, which have been recognised to be difficult for consumers to interpret^(8)^. On an international level, more than forty countries use some sort of government-endorsed FOP nutritional labelling scheme on selected food products^(9)^. For instance, positive endorsement logos are only displayed on products that fulfil certain nutritional criteria with a healthier nutrient profile than others in the same food category, while some labels are based on rating schemes, which grades the overall healthiness of a product^(9)^.

Nutrient profiling labelling schemes vary in several ways, for instance, in terms of presentation, communicated health messages and nutrient focus. Most FOP nutrition labels focus on nutrients such as Na, SFA and trans-fatty acids, and added, free, or total sugars^(10)^, all of which are of concern for diet-related non-communicable diseases. The labelling schemes may also include positive components of food items and diets, such as fibre and wholegrains, as well as fruits and vegetables. This type of nutrition labelling supports a healthier diet primarily as it guides the consumer to make informed food choices^(6)^.

In 1989, The Swedish Food Agency established the endorsement logo the Keyhole^(11)^. The Keyhole criteria were revised in 2021^(12)^, and the symbol has a current recognition factor of 97 %^(13)^. The aim of the Keyhole is to guide consumers to make healthier food choices using a criteria-based nutrition label that is in line with the Swedish dietary guidelines and the Nordic Nutrition Recommendations^(14)^, with specific criteria for different food groups. The Keyhole symbol is used to highlight the healthier options within a food group, targeting nutritional composition of total fat, SFA, trans-fat, salt, total or free sugars, and dietary fibre, as well as the content of wholegrains, fruits and vegetables including legumes. Additionally, it may be used to label healthy unprocessed foods, such as vegetables, fruits and fish^(12)^. The symbol is available for core foods, such as vegetables, fruits, fish, cereal and cereal products, dairy products, and meat, and also for ready meals. Discretionary products such as cakes, sweets and soft drinks cannot carry the logo. The health logo is voluntary and free of charge and can be used by manufacturers if the nutritional criteria are met. Today, the Keyhole is used in Sweden, Denmark, Norway, Iceland, Lithuania and North Macedonia^(10)^. In addition to guiding the consumers, FOP nutrition labels aims to drive reformulation and more healthy product development^(6)^. The Dutch Choices logo, another positive FOP label, shares many characteristics with the Keyhole logo^(10)^. A recent study of the Choices logo brought novel evidence that food labelling had incentivised reformulation. The study demonstrated that compared to general food items on the market, healthier labelled food items were more likely to have been reformulated in the previous 10 years^(15)^.

Studies on the nutritional impact of shifting the diet to meet FOP nutrition labelling schemes have investigated hypothetical scenarios in adults. One previous study examined the potential impact of the Keyhole on nutrient intake of Swedish adults using a weekly menu created based on food consumption statistics^(14)^. Foods that were non-compliant to the Keyhole criteria in the weekly menu were replaced with compliant food alternatives, resulting in improved intakes of SFA, added sugars, dietary fibres and wholegrain. However, unintentional reductions were seen in intakes of MUFA, PUFA and in energy. Studies on the impact of other FOP nutrition labels have generally demonstrated improvements in the diet for the particular nutrients of concern in the labelling schemes^(16–20)^, although in one study negative effects on intakes of fat-soluble vitamins were observed^(16)^. The potential impact on nutritional quality of replacing foods currently consumed with foods meeting the Keyhole nutritional criteria has, to our knowledge, not yet been investigated in children and adolescents, nor has the impact of the Keyhole symbol on micronutrient intake been reported.

Health promotion efforts through FOP nutrition labels can give guidance to a healthier diet for adolescents, as well as adult consumers. The Keyhole is a well-recognised and established example of this in Sweden, which is often integrated into early educational curricula. The present study aimed to investigate the potential impact of the Keyhole on adolescents’ nutrient intake by modelling a shift from reported dietary intakes to foods meeting the Keyhole nutritional criteria, using dietary data from a Swedish national dietary survey of adolescents.

## Methods

### Study design and population

Dietary data were derived from Riksmaten Adolescents 2016–2017, a cross-sectional school-based dietary survey in Sweden. A nationally representative sample of Swedish adolescents in school years 5, 8 and 11 was recruited, with mean ages 12, 15 and 18 years, respectively. The survey was conducted by the Swedish Food Agency, and details of the study design and recruitment are described by Moraeus *et al.* ^(21)^. In summary, schools were randomly selected from the national school register with sampling based on school size, geographic area and municipality characteristics. These schools were then invited to participate, and the study was conducted in 1–2 classes within participating schools. Trained staff from the Swedish Food Agency visited the classes to instruct pupils on how to report their dietary intake in a web-based system and to collect anthropometric data. Height and weight were measured with standardised equipment. Both the participants and their legal guardians completed questionnaires on background data, for example, parental education level. Of 5145 pupils invited, the 3099 participants with full dietary information from 2 d of retrospective registration were included in the present study. The participants were representative of the Swedish population regarding socio-economic background and school organisation, and participating schools covered geographical areas across the country with all municipality types represented^(21)^.

### Dietary assessment

Dietary intake was assessed with the web-based dietary assessment method ‘RiksmatenFlexDiet’, a biomarker-validated dietary assessment method comparable to the 24-h recall method^(22)^. With RiksmatenFlexDiet participants registered their food intake retrospectively on two non-consecutive days, recalling what they had consumed the day before registration. The first recall day was the day before the school visit and was registered at school. The second recall day was randomly assigned to 2–7 subsequent days and could be registered in any location^(21)^. The participants registered their intake from a food list of 778 foods and drinks adapted for the study population, by selecting foods consumed and specifying intake amounts, either as standard portions sizes, pieces, household measurements or through portion pictures. Before submitting the record, probing questions about easily forgotten foods were asked, and in a final step, participants reviewed food intake amounts and eating occasions. It took about 15–30 min to register one day’s intake, depending on the age of the participant^(22)^. The food list was linked to the Swedish Food Agency’s food composition database, version Riksmaten Adolescents 2016–2017, allowing for direct calculation of energy and nutrient intake. Content of free sugars was calculated according to the systematic method described by Wanselius *et al.* ^(23)^ Assessment was from September to May with registration days were evenly distributed across the week^(21)^.

### Selection of replacement foods

To investigate the potential impact of eating in accordance with the Keyhole nutritional criteria on adolescents’ nutrient intake, hypothetical food replacements were performed based on the adolescents’ reported food intakes. Non-compliant foods were replaced with comparable foods complying with the Keyhole criteria when possible. Replacements were performed on foods as consumed, hence not including ingredients in recipes. Food groups included were dairy products (e.g. milk, fermented milk products and cheese), cereal products (e.g. bread, breakfast cereals, pasta and rice), meat products and alternative vegetarian products (e.g. sausages and cold cuts), and fats (e.g. fat spreads and cooking fats). Food replacements were simulated at varying levels on a product-by-product basis, substituting a non-compliant food item with a similar item within the food group that complied with the criteria, for example, a non-compliant flavoured yogurt was replaced with a flavoured yogurt that complied with the criteria. Keyhole eligible food groups were excluded in the simulation if they fulfilled the Keyhole nutritional criteria per se, or when we did not have enough information to make replacements. Food groups that fulfilled the criteria by themselves were vegetables, fruits, and unprocessed nuts, fish, shellfish and poultry; consequently, there were no need for replacements within these groups. For some food groups, the participants had limited options to choose from when recording their food intakes in regard to the Keyhole criteria (processed fishery products, unprocessed meat, pieces of meat, minced meat, sauces and condiments). Thus, there were not enough variation in the food list to conclude whether the consumed item complied with the criteria or not. There are also Keyhole criteria for ready meals, but these foods could not be distinguished from other dishes in the food list and were therefore excluded.

The food composition data for the foods included in the Riksmaten Adolescents 2016–2017 survey food list were compared to the Keyhole nutritional criteria^(12)^ for the selected food categories.

Food replacements were taken from the following sources:

- Swedish Food Agency food composition database, version Riksmaten Adolescents 2016–2017 (food items already included in the survey food composition database);
- if not applicable, from the national Swedish Food Agency food composition database version 20200116;
- or, in a few cases where no replacement alternative was available; a new food item was added based on Keyhole-labelled alternatives available on the market.

The choice of replacement food was the alternative closest to target nutrient levels of Keyhole nutritional criteria per food group, that is, just meeting the criteria, not necessarily the best available option regarding the nutrients of concern. For the added items, nutrient levels just meeting the criteria levels were also chosen. The supplemental table shows examples of food replacements in each included food group with corresponding nutritional criteria.

### Food replacement scenarios

Replacements were made in each participant’s food intake when non-compliant foods were reported to have been consumed, simulating diets that included more Keyhole compliant foods than the reported intakes. This was done by first adding the Keyhole compliant food items with nutritional information into the food list and thereafter recalculating the dietary intakes of the adolescents.

To explore nutritional effects of varying diet shifts, replacement scenarios were modelled at differing levels of compliance to Keyhole foods. The rationale was that it is unlikely that everyone would completely replace all parts of their diet. Effects on nutrient, energy and wholegrain intakes were analysed in different replacement scenarios described below.

- 50 % replacement was a partial shift to Keyhole-compliant foods. All reported non-compliant foods were included but substituted by 50 %, while 50 % were kept the same (i.e. half of the weight of each reported food item, respectively, was replaced), for example, in practice meaning that you would replace every other bowl with cereal not meeting the criteria.
- 100 % replacement comprised full replacement of all participants’ reported non-compliant foods to Keyhole-compliant alternatives.
- 100 % replacement energy-corrected indicated full replacement of all participants’ reported non-compliant foods but also applying an energy correction factor to hold the energy intake constant, as energy intake tended to decrease with the replacement scenarios. For each replacement food item, an energy correction factor matched the energy content of the reported item, thereby adjusting portion sizes. In this energy-corrected scenario, participants were assumed to compensate for any changes in energy intake by changing intake amounts of the replacing food items.
- Additionally, two more scenarios were modelled in which food items were partially replaced to an extent of 25 % and 75 % to investigate the proportion of adolescents who would meet nutrition recommendations for the Keyhole-regulated nutrients and components (SFA, MUFA, PUFA, free sugars, dietary fibre, wholegrains and salt).

To understand whether reported and simulated intakes complied with dietary recommendations, Nordic Nutrition Recommendations were used as reference^(24)^. When there were no established Nordic recommendations, European Food Safety Authority’s dietary reference values were used for micronutrients^(25)^. WHO’s recommendations were used for free sugars^(26)^. Wholegrain intakes were compared to Swedish Food Agency’s recommendations^(27)^. For the Keyhole-regulated components, intakes were considered to meet recommendations if: SFA < 10 % of total energy (E %); MUFA 10–20 E %; PUFA 5–10 E %^(24)^; free sugars <10 E %^(26)^; and wholegrains ≥7·5 g/MJ^(27)^. Intakes of dietary fibre were considered to meet recommendations if >2 g/MJ for all participants. Salt intakes were considered to meet recommendations if not exceeding the population nutrient goal of 6 g/d^(24)^.

### Statistical methods

To estimate average intakes of energy, nutrients and wholegrains per individual, the mean intake over 2 d was calculated per participant. As intake distributions were skewed, population intakes are expressed in medians (p50) and interquartile ranges (p25; p75). Wilcoxon signed rank test was used to evaluate differences between the reported intakes and the scenario intakes. Logistic regression analysis was computed to explore if certain sociodemographic factors were associated with greater nutritional impact of a shift towards adherence to Keyhole nutritional criteria for the uncorrected 100 % scenario. This was performed on components included in the Keyhole criteria (SFA, MUFA, PUFA, dietary fibre, free sugars, salt and wholegrains). The dependent variable in each model was coded as meeting nutrition recommendations in the scenario but not in reported intakes, *v*. no benefit from the simulation. No benefit refers to no change in nutrition recommendation compliance between reported or scenario intakes or in a few cases changing to non-compliance levels. Independent variables in the models were sex, school year, BMI status according International Obesity Task Force cut-offs^(28)^, highest attained parental education level and school municipality size according to Swedish Association of Local Authorities and Region’s classification^(29)^. The logistic regression analysis was carried out stepwise, first with full models and thereafter with models including statistically significant variables. Statistical significance level was set at <0·01 to account for multiple testing. Data modelling and statistical analyses were performed using Stata version 16.1. (StataCorp. 2019. Stata Statistical Software: Release 16. StataCorp LLC).

## Results

### Description of participants

Table 1 describes the characteristics of the study sample. A total of 3099 adolescents completed the survey, with a participation rate of 60 %.

**Table 1:** Characteristics of 3099 participating adolescents

	*n*	%
Sex		
Girls	1710	55
Boys	1389	45
School year		
5 (Mean age 12 years)	1049	34
8 (Mean age 15 years)	1050	34
11 (Mean age 18 years)	1000	32
BMI status*		
Normal weight or underweight	2426	78
Overweight including obese	646	21
Missing	27	1
Parental education level†		
≤12 years	1124	36
>12 years	1781	57
Missing	194	6
School municipality size‡		
Large cities	896	29
Medium-sized towns	1313	42
Smaller towns	890	29

* Determined according to International Obesity Task Force’s age-and sex-adjusted cut-off points.

† Determined as the highest attained education level of either parent.

‡ Determined according to classification by Swedish Association of Local Authorities and Regions.

### Replacing food items

**Fig. 1:**
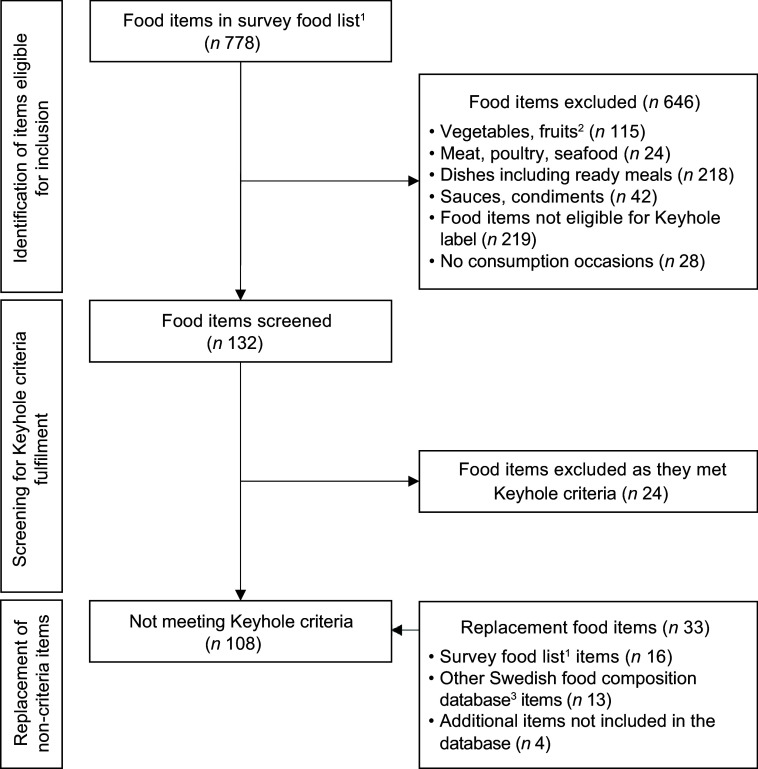
Flow of identifying and replacing food items in the modelled scenarios (*n* 778, food items excluded = 670). 108 items in the survey food list were replaced with 33 similar food items complying with the Keyhole nutritional criteria. ^1^Swedish Food Agency food composition database, version Riksmaten Adolescents 2016–2017. ^2^Also including legumes, berries, mushrooms and unprocessed nuts. ^3^Swedish Food Agency food composition database, version 20200116

A total of 108 foods non-compliant with the Keyhole nutritional criteria were replaced with a total of thirty-three similar foods that complied, as illustrated in Fig. 1.

Of all foods registered during recall days, 31 % could be replaced with Keyhole alternatives. Most commonly, full-fat dairy was replaced by reduced fat alternatives, refined cereal products were substituted with products higher in wholegrains, and fat spreads and blends were replaced by substitutes with a healthier fatty acid composition. Meat products were replaced with lower-fat alternatives, including substitution of pork sausages and cold cuts with compliant poultry products available in the food composition database.

### Change in daily nutrient intakes

Table 2 shows reported and simulated median daily intakes of Keyhole-regulated nutrients and components (total fat, SFA, MUFA, PUFA, dietary fibre, free sugars, salt and wholegrain), and energy, protein, and carbohydrates. Reported intakes were compared to scenarios simulated at 50 %, 100 % and energy-corrected 100 % replacement. Differences between reported intakes and scenario intakes were seen for all nutrients and components (except when equivalent, by definition). Improved intake changes were observed for SFA, PUFA, dietary fibre, wholegrains, free sugars and salt, while negative changes were observed for MUFA.

**Table 2:** Median daily energy, nutrient and wholegrain intakes of 3099 adolescents in Sweden participating in the national survey Riksmaten Adolescents 2016–2017 (reported intake) and in intake scenarios where foods were replaced to meet the Keyhole nutritional criteria

Nutrient/component	Reported intake		50 % replacement		100 % replacement		100 % replacement energy-corrected	
	Median	p25, p75	Median	p25, p75	Median	p25, p75	Median	p25, p75
Energy, kJ	8338	6598, 10 597	8194	6481, 10 440	8070	6358, 10 269	8338*	6598, 10 597
Total fat, E %	34	30, 39	33	29, 38	32	28, 36	32	28, 36
SFA, E %	13	11, 16	12	11, 14	11	9, 13	11	10, 13
MUFA, E %	13	11, 15	13	10, 15	12	10, 14	12	10; 14
PUFA, E %	4·4	3·6, 5·4	4·9	4·0, 6	5·4	4·3, 6·8	5·3	4·3, 6·6
Protein, E %	16	14, 19	17	15, 20	18	15, 21	18	16, 21
Carbohydrates, E %	47	42, 51	47	43, 52	48	43, 52	47†	42, 51
Dietary fibre, g/MJ	2·0	1·6, 2·5	2·2	1·8, 2·7	2·3	1·9, 2·9	2·3	1·9, 2·8
Wholegrains, g/MJ	2·4	1·0, 4·8	5·3	3·1, 8·2	8	4·7, 12	7·6	4·5, 11·4
Free sugars, E %	11	6·6, 16	10	6·2, 15	10	5·8, 15	10	5·7, 15
Salt, g	7·8	6·0, 10	7·6	5·9, 10	7·5	5·8, 9·8	7·8†	6·0, 10

* Equal to reported intake by definition.

† Minor intake increases that were statistically significant are not visible due to rounding.

In the intake scenarios, foods were replaced to meet the Keyhole nutritional criteria at 50 %, 100 % and 100 % with an energy correction factor applied to avoid a reduction in total energy intake. All replacement scenario intakes were statistically significantly different from reported intakes (Wilcoxon signed rank test, *P* < 0·001), not applicable for identical modelled energy intake levels in scenario 100 % replacement energy-corrected.

**Fig. 2:**
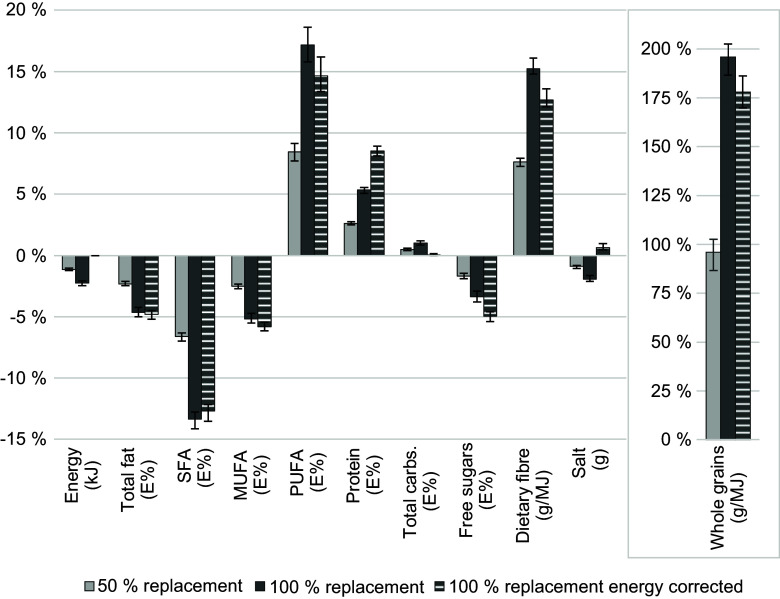
Percentage change in median daily energy, nutrient and whole grain intakes in adolescents when replacing foods to meet the Keyhole nutritional criteria in modelled scenarios compared to reported intakes assessed in Riksmaten Adolescents 2016–2017 (*n* 3099). Error bars represent 99 % CI. SFA, saturated fatty acids; MUFA, monounsaturated fatty acids; PUFA, polyunsaturated fatty acids; carbs., carbohydrates

Figure 2 shows the percentage change in median daily intakes of the same nutrients and components for the three replacement scenarios compared to the reported intakes, corresponding to values shown in Table 2. Energy intakes were reduced in the 50 % and 100 % replacement scenarios (-1·1 % and -2·3 %, respectively). Reductions were also seen in all three replacement scenarios for intakes of total fat, SFA, MUFA and free sugars (median reductions ranging from maximum -13 % for SFA in the 100 % replacement scenario to minimum -1·7 % for free sugars in the 50 % replacement scenario). Median salt intakes were reduced in the 50 % and 100 % replacement scenarios (-0·9 % and -1·9 %) but slightly increased in the energy-corrected scenario (+0·6 %). Increases were observed in all three scenarios for intakes of PUFA, protein and dietary fibre (median increases ranging from maximum +17 % for PUFA in the 100 % replacement scenario to minimum +2·6 % for protein in the 50 % replacement scenario). Minor increases were seen in carbohydrate intakes, a median increase of 1 % in the 100 % replacement scenario. A large increase in wholegrain intakes was seen in the three scenarios, where median intake levels increased with almost 200 % in the 100 % replacement scenario.

**Fig. 3:**
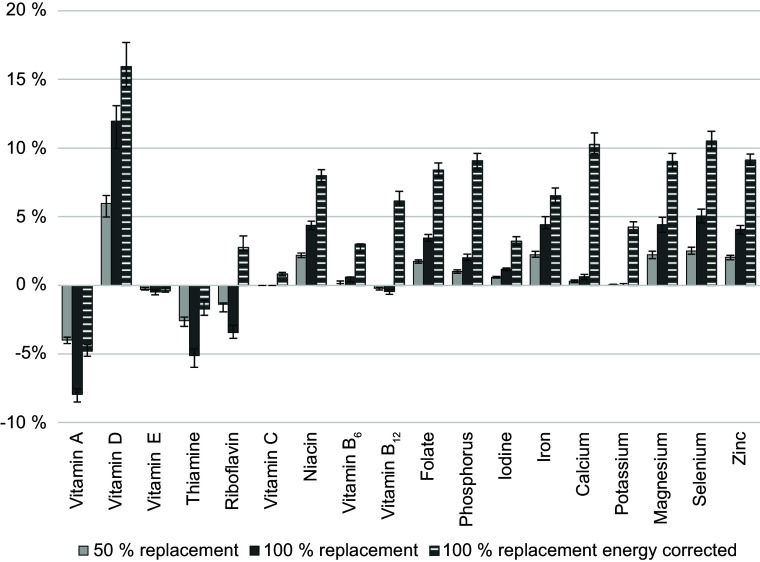
Percentage change in median daily micronutrient intakes in adolescents when replacing foods to meet the Keyhole nutritional criteria in modelled scenarios compared to reported intakes assessed in Riksmaten Adolescents 2016–2017 (*n* 3099). Error bars represent 99 % CI

Figure 3 shows the percentage change in median daily micronutrient intakes for the 50 %, 100 % and energy-corrected 100 % replacement scenario compared to the reported intakes. Micronutrients included vitamin A, vitamin D, vitamin E, thiamine, riboflavin, vitamin C, niacin, vitamin B_6_, vitamin B_12_, folate, P, I, Fe, Ca, K, Mg, Se and Zn. The largest differences observed were in vitamin D intakes, where median intakes were increased between 6 % and 16 % in the three scenarios. Increases in median daily intakes were most pronounced in the energy-corrected 100 % scenario and seen for riboflavin, vitamin C, niacin, vitamin B_6_, vitamin B_12_, folate, P, I, Fe, Ca, K, Mg, Se and Zn, while being unaffected or nearly unaffected in the uncorrected scenarios. Decreased median intakes of vitamin A and thiamine were seen in the three scenarios, also decreased intakes of riboflavin and a minor decrease in vitamin E intakes in the uncorrected scenarios. In summary, other than the negative changes in intakes of vitamin A, thiamine and riboflavin, median micronutrient intakes were improved or stable.

### Nutrition recommendations comparisons

**Fig. 4:**
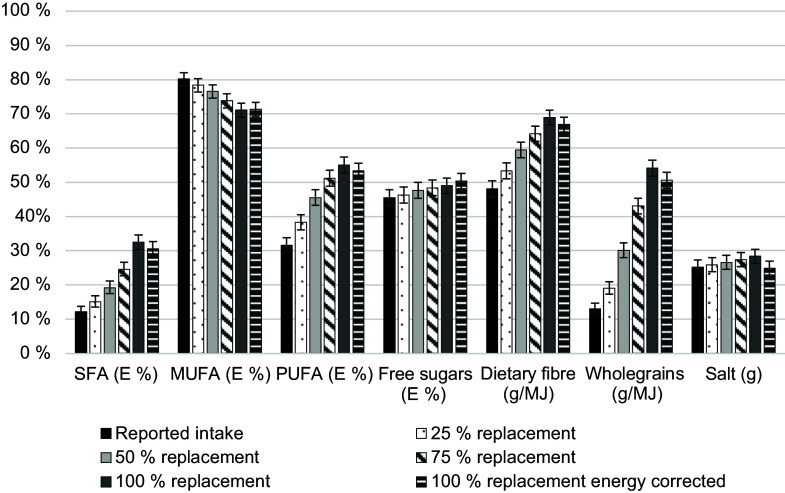
Percent of adolescents (*n* 3099) in compliance with macronutrient, whole grain and salt recommendations in Riksmaten Adolescents 2016–2017 (reported intake) and in intake scenarios where foods were replaced to meet the Keyhole nutritional criteria. Error bars represent 99 % CI. SFA, saturated fatty acids; MUFA, monounsaturated fatty acids; PUFA, polyunsaturated fatty acids. Intakes of SFA, MUFA, PUFA, dietary fibre and salt were compared to the Nordic Nutrition Recommendations 2012, free sugars to World Health Organization’s recommendations, and whole grains to Swedish Food Agency’s recommendations

Intakes of the Keyhole-regulated nutrients and components (SFA, MUFA, PUFA, free sugars, dietary fibre, wholegrains and salt) in the 25 %, 50 %, 75 %, 100 % and energy-corrected replacement scenarios were compared to nutrition recommendation compliance levels (Fig. 4). The proportion of participants meeting the recommendation increased with the increase in food replacement for SFA, PUFA, free sugars, dietary fibre, wholegrains and salt, although a decrease was seen in participants meeting the recommendation for MUFA. When correcting the 100 % scenario for energy, the effects were less pronounced than in the uncorrected 100 % scenario for SFA, PUFA, dietary fibre and wholegrains. However, with energy correction, a higher proportion met the recommendation for free sugars intake and the negative effect on MUFA intake was somewhat reduced. The proportion of participants complying with salt recommendations was not improved in the energy-corrected scenario; however, differences from reported salt intakes were small in all scenarios.

To demonstrate potential improvements on micronutrient intake of a shift towards Keyhole foods at different levels of food replacement, percent of adolescents complying with requirements is viewed in a supplemental Figure. Note that calculations are based on mean intakes from 2-d registrations, not usual intakes. Overall, more adolescents met the nutrient requirements when simulating dietary replacements, although negative changes were observed in the uncorrected 100 % scenario for vitamin A and thiamine.

When analysing whether the simulated impact differed between sociodemographic groups according to sex, school year, parental education level, school municipality size and BMI for the Keyhole-regulated components, only differences between school year and sex were statistically significant. Results of the statistically significant variables are presented in Table 3. Participants in the lower school years were 35–41 % more likely to shift from not meeting nutrition recommendations in the reported intakes to meeting recommendations in the food replacement scenario for SFA, PUFA, dietary fibre and wholegrains compared to the older adolescents. Likewise, girls would be more likely than boys to correspond to salt recommendations when simulating a shift towards Keyhole foods. However, although the relative salt effect was big between sexes (girls were 78 % more likely to comply with salt recommendations than boys), the increased compliance was small (2·4 % for boys and 4·2 % for girls). No differences were observed regarding intakes of MUFA or free sugars.

**Table 3:** Group differences in increased compliance with nutrition recommendations when replacing foods not meeting the Keyhole nutritional criteria with Keyhole-compliant alternatives in the 100 % replacement scenario (*n* 3099). Only variables with significant associations are presented

Variables	Increased compliance, proportion (%)	99 % CI	Odds ratio*	99 % CI	*P*
SFA					
School year					
Year 11 (reference)	18	15, 21	1		
Year 8	20	17, 23	1·13	0·85, 1·52	ns
Year 5	23	20, 27	1·40	1·05, 1·86	0·002
PUFA					
School year					
Year 11 (reference)	24	20, 27	1		
Year 8	25	21, 28	1·06	0·81, 1·38	ns
Year 5	29	26, 33	1·35	1·04, 1·75	0·003
Dietary fibre					
School year					
Year 11 (reference)	18	15, 22	1		
Year 8	21	18, 24	1·20	0·90, 1·60	ns
Year 5	23	20, 27	1·36	1·03, 1·81	0·005
Wholegrains					
School year					
Year 11 (reference)	36	32, 40	1		
Year 8	43	39, 47	1·37	1·09, 1·73	<0·001
Year 5	44	40, 48	1·41	1·12, 1·78	<0·001
Salt					
Sex					
Boys (reference)	2·4	1·5, 3·7	1		
Girls	4·2	3·1, 5·6	1·78	1·03, 3·09	0·007

* OR for an increase in compliance with nutrition recommendations.

Logistic regression analysis was carried out on components included in the Keyhole criteria (SFA, MUFA, PUFA, dietary fibre, free sugars, wholegrains and salt). Full models included sex, school year, BMI status, highest attained parental education level and school municipality size.

## Discussion

The Keyhole logo is a FOP nutrition label guiding healthy food choices by targeting food composition of dietary fats, sugars, fibre, wholegrains and salt^(12)^. Results from this theoretical modelling study, where foods not complying with the Keyhole nutritional criteria were replaced with compliant alternatives, generally showed a shift into the direction of the nutrition recommendations. This was generally observed for Keyhole-regulated nutrients as well as micronutrients not included in the criteria, and even when making partial replacements in the adolescents’ diets. However, a few unintentional effects in nutrient intakes were also observed.

For those nutrients and components that are regulated by the Keyhole criteria, intakes were mostly improved in modelled scenarios, but with one exception in the case of MUFA intake. This was due to the decrease in total fat content and a shift from MUFA- to PUFA- containing foods containing foods, largely affected by replacements of butter and oil blends to margarine. However, improved intake changes were seen for both PUFA and SFA, which resulted in overall beneficial fat composition intakes in the replacement scenarios compared to the reported intakes. For the other macronutrients, total carbohydrates were mainly stable in terms of percent of total energy with only minor intake increases in the scenarios as compared to reported intakes, while energy-percent protein intakes were increased. This was mainly due to the move from refined cereal products to wholegrain products, replacement of full-fat cheese with low fat cheese and the shift from high- to lower-fat protein-rich animal-based products. Both fibre and wholegrain intakes were improved, with a notable increase in wholegrain intakes when cereal products containing little or no wholegrain were replaced with wholegrain products. Intake reductions of free sugars were relatively small, which can be explained by the fact that most types of food containing substantial amounts of free sugars cannot carry the Keyhole label. The relatively modest changes in salt intakes can be attributed the fact that mixed dishes, the food group that contributed with most salt in adolescents’ diets, were not included in the simulation and therefore not affected.

Concerning micronutrient intakes, we observed that most were stable or improved in the replacement scenarios. Generally, most Swedish adolescents have adequate intakes of micronutrients, although intakes of Fe, vitamin D and folate may be of concern in some groups of adolescents^(5)^. The results from the modelling showed that intakes of these micronutrients were improved. However, some negative effects were observed, most apparent for median intakes of thiamine and vitamin A. For thiamine, a change from fortified breakfast cereals to muesli made the greatest difference on the intake reduction, together with replacements of pork-based meat products with poultry-based alternatives. Replacement of higher-fat dairy products with low-fat alternatives had the greatest impact on the vitamin A intake reduction, as milk is not fortified with vitamin A in Sweden. The increase in vitamin D intakes was a result of substituting high-fat dairy products with lower/healthier fat alternatives that were fortified with vitamin D. It should be noted that recent increases in mandatory vitamin D fortification in Sweden^(30,31)^ might leave less room for potential improvement than those suggested in our simulation of earlier food composition data. Compared with the older vitamin D regulations, more foods now require fortification and with higher contents of vitamin D in Sweden.

Total energy intake will be reduced if adolescents shift their diets towards Keyhole-labelled foods without compensating for lower energy density in the diet by eating more. This is mainly explained by the decrease in total fat intake. However, even as the energy intake was reduced in the uncorrected scenarios, most nutrients were unaffected or even improved. Our results imply that shifting the food intake towards Keyhole-labelled foods can be helpful for healthy weight regulation. While the Keyhole does not aim to lower energy intake in children or in adults, findings of a drop in energy intake are consistent with estimations in adults when replacing foods in current diets with Keyhole-labelled foods^(14,32,33)^. Although potentially beneficial in some individuals, it is possible that the reduction in energy intake could result in compensatory eating of other unhealthy foods.

When comparing the simulated intakes to the nutrition recommendations, there were improvements in intakes of SFA, PUFA, dietary fibre and wholegrains even at low proportions of food replacement. For free sugars and salt, potential improvements in diet were less pronounced. Girls benefited more than boys in terms of salt intakes, and younger adolescents benefited more than the older adolescents for SFA, PUFA, dietary fibre and wholegrains. However, no other group difference in associations were seen, which is in line with results from other studies modelling impacts of endorsement logos in adults, showing minor or comparable effects between groups^(16,18)^. Still, many adolescents did not comply with the recommendations even in the 100 % replacement scenario. Potential improvements in nutrient intake from endorsement logos are dependent on the original food intake and the proportion of foods available for replacement. For instance, if a high proportion of foods consumed are discretionary and other ineligible foods or foods already complying with nutritional criteria, there will not be much room for improvement. As we did not include all foods eligible for the Keyhole, the effect of a shift to Keyhole foods could potentially be larger than those simulated here. However, many popular foods are not eligible for labelling, and shifting the diet to Keyhole foods cannot be the sole solution to achieve nutrition recommendations, particularly adolescents who consume a high proportion of discretionary foods^(2)^ that are ineligible for labelling.

To benefit from endorsement logos, a certain level of knowledge and understanding is needed as otherwise these endorsement-labelled foods may be misinterpreted as being generally healthy, rather than relatively healthier within a given food category^(34)^ or even imply a licence to overconsume excess amounts of food. A recent study in British adults examined the effectiveness of consumer ability to identify healthiness of foods based on five different FOP nutrition labelling systems of which one was an endorsement logo^(35)^. When comparing the different FOP labels, the endorsement logo had a lower effect than the summary indicator Nutri-Score^(36)^ which had the largest effect, yet all labels were more effective than no label^(35)^.

Real-life evidence of the nutritional impact of FOP nutrition labels on diet is limited and required consideration of consumer understanding. Moreover, existing evidence that understanding positive logos is associated with dietary behaviour cannot imply causality^(37)^. In contrast, modelling studies are a useful alternative to estimate possible nutritional impact of diet replacements on consumers’ diets, that is, predict the nutritional consequences of a public health strategy. However, modelling studies may be criticised as only revealing dietary improvements under ideal circumstances, as simulations tend to be more ambitious than what food purchasing studies suggest^(9)^. To meet this criticism and model reasonably realistic situations, various scenarios were created from adolescents’ self-reported food intakes to demonstrate nutritional impacts that are lower than maximal. Moreover, when choosing food replacements, we were conservative and did not opt for the best available option but rather the food items closest to target nutrient levels of Keyhole nutritional criteria.

This study is not without other limitations. As in other dietary surveys where misreporting occurs due to, for example, unawareness of one’s food intake and social desirability, this type of reporting error is also probable in the present study and may contribute to biases in our simulated results. Moreover, the limited number of replacing food items, in this study, may have resulted in unrealistic food replacements. Finally, it is acknowledged that this type of study cannot account for highly relevant factors such as product availability and reformulation, affordability, knowledge, or personal preferences that may drive choices of logo foods. If the proportions of adolescents complying with nutrition recommendations in Riksmaten Adolescents are overestimated, then the potential improvement by shifting to Keyhole-labelled foods would be greater than the simulation in this study indicates.

In summary, by simulating a partial shift from adolescents’ reported diets towards Keyhole-labelled foods, overall nutrient intakes were improved even with minor proportions of dietary replacements. If not compensated with greater amounts of foods, energy intakes were decreased by the food replacements, although without any considerable negative effects on nutrients except possible lowered vitamin A intakes. The results suggested a potential for adolescents to greatly increase wholegrain intakes, while only slight improvements could be observed in intakes of free sugars and salt. We conclude that this theoretical simulation shows that a shift to Keyhole alternatives of everyday foods would improve adolescents’ nutritional intake. Thus, using the Keyhole as a public health strategy may guide the choice of alternatives for core foods and dishes as dairy products, fat and cereal products towards healthier nutrient intakes. However, further health promotion efforts beyond Keyhole-labelled foods would be needed to help adolescents limit their intakes of sugar and salt from discretionary foods and drinks.

## Supplementary material

For supplementary material accompanying this paper visit https://doi.org/10.1017/S1368980022002178. click here to view supplementary material
