# Supplementary material for: Consumption of foods with the Keyhole front-of-pack nutrition label: potential impact on energy and nutrient intakes of Swedish adolescents
Source: Public Health Nutr. 2022 Sep 30;25(12):3279–90. doi: 10.1017/S1368980022002178 (PMC9991761; doi:10.1017/S1368980022002178)
Supplement: Supplementary file 1 [file S1368980022002178sup.zip › S1368980022002178sup002.docx]

|  | Reported intake | 50% replacement | 100% replacement | 100% replacement energy corrected |
| --- | --- | --- | --- | --- |
| Vitamin A | 57.0244% | 53.9024% | 48.9756% | 52.7317% |
| Vitamin D | 24.8145% | 29.0416% | 33.2365% | 36.9151% |
| Vitamin E | 90.5131% | 90.6099% | 90.3195% | 90.4808% |
| Thiamine | 66.4878% | 63.6585% | 58.8293% | 63.6585% |
| Riboflavin | 66.0976% | 63.9512% | 61.4146% | 68.1951% |
| Vitamin C | 57.4146% | 59.9024% | 61.6098% | 64.3415% |
| Niacin | 98.0316% | 98.4511% | 98.7093% | 98.9029% |
| Vitamin B6 | 82.7805% | 83.0244% | 82.7317% | 85.2683% |
| Vitamin B12 | 95.0307% | 94.6112% | 94.1271% | 95.2243% |
| Folate | 70.1463% | 72.2927% | 73.9512% | 77.4146% |
| Phosphorus | 99.1933% | 99.3224% | 99.3869% | 99.516% |
| Iodine | 93.4495% | 93.7722% | 93.9658% | 94.5144% |
| Iron | 46.3779% | 48.7008% | 50.6693% | 52.7953% |
| Calcium | 87.8348% | 87.6412% | 87.0926% | 89.5127% |
| Potassium | 100% | 100% | 100% | 100% |
| Magnesium | 50.0807% | 53.4689% | 56.4698% | 61.2778% |
| Selenium | 61.0519% | 64.0207% | 66.1504% | 71.4747% |
| Zinc | 92.6751% | 93.4495% | 94.4498% | 95.5147% |

**Supplemental figure.** Percent of adolescents (n=3099^1^) who complies with micronutrient requirements Riksmaten Adolescents 2016-17 (reported intake) and in intake scenarios where foods were replaced to meet the Keyhole nutritional criteria, based on median intakes of two days registration.

Nutrient intakes were compared to nutrient requirements for age group and/or sex respectively. The average requirements (AR) from the Nordic Nutrition Recommendations 2012 (NNR 2012) were used for all micronutrients except for potassium and magnesium where no AR values are defined in the NNR 2012, instead adequate intake levels from the European food safety authority were used. Error bars represent 99% CI.

^1^For vitamin A, thiamine, riboflavin, vitamin C, vitamin. B6, folate and iron, AR values are not defined for all population groups in the NNR 2012. Only the participants in the population groups with AR values defined were included in analysis.
