# Supplementary material for: Consumption of foods with the Keyhole front-of-pack nutrition label: potential impact on energy and nutrient intakes of Swedish adolescents
Source: Public Health Nutr. 2022 Sep 30;25(12):3279–90. doi: 10.1017/S1368980022002178 (PMC9991761; doi:10.1017/S1368980022002178)
Supplement: Supplementary file 1 [file S1368980022002178sup.zip › S1368980022002178sup001.docx]

**Supplemental table**. Keyhole nutritional criteria for included food groups and examples of replacements

| **Food group** | | **Keyhole nutritional criteria** | | **Examples of reported food items** | **Examples of replacing food items** |
| --- | --- | --- | --- | --- | --- |
| Dairy products | |  |  |  |  |
|  | Milk, unflavoured | Total fat | ≤0.7 g/100 g | Full fat milk | Skimmed milk (0.5%) |
|  | Fermented milk products, unflavoured | Total fat | ≤1.5 g/100 g | Natural yoghurt (3%) | Natural light yoghurt (0.5%) |
|  | Fermented milk products, flavoured | Total fat  Free sugars | ≤1.5 g/100 g  ≤4.0 g/100g | Full fat flavoured yoghurt | Flavoured yoghurt (1.5%) |
|  | Products comprising a mixture of milk and cream, unflavoured | Total fat | ≤5.0 g/100 g | Crème fraiche | Light crème fraiche (5%) |
|  | Cheese | Total fat  Salt | ≤17 g/100 g  ≤1.6 g/100g | Hard cheese (27%) | Hard cheese (17%) |
|  | Fresh cheese | Total fat  Salt  Free sugars | ≤5.0 g/100 g  ≤0.9 g/100 g  ≤1.0 g/100g | Cream cheese (27%)  Feta cheese | Cream cheese extra light (5%)  Light cheese (tofu) |
| Cereal products | |  |  |  |  |
|  | Rice | Dietary fibres  Whole grains | ≥6.0 g/100g  100% | White rice | Whole grain brown rice |
|  | Breakfast cereals | Total fat  Total sugars  Free sugars  Dietary fibres  Salt  Whole grains | ≤8.0 g/100 g  ≤13 g/100 g  ≤9.0 g/100 g  ≥6.0 g/100 g  ≤ 1.0 g/100 g  ≥55% | Granola  Corn flakes | Whole grain fruit muesli  Whole grain fruit muesli |
|  | Porridge | Total fat  Total sugars  Dietary fibres  Salt  Whole grains | ≤4 g/100 g  ≤5 g/100 g  ≥1 g/100 g  ≤0.3 g/100 g  ≥55% | Rice porridge | Oatmeal porridge |
|  | Soft breads | Total fat  Total sugars  Dietary fibres  Salt  Whole grains | ≤7 g/100 g  ≤5 g/100 g  ≥5 g/100 g  ≤1.0 g/100 g  ≥30% | White soft bread, refined  Brown soft bread | White soft bread with whole grains  Brown soft bread, meeting sugars criterion |
|  | Hard breads | Total fat  Total sugars  Dietary fibres  Salt  Whole grains | ≤7 g/100 g  ≤5 g/100 g  ≥6 g/100 g  ≤1.3 g/100 g  ≥50% | White hard bread | Whole grain hard bread |
|  | Pasta | Dietary fibres  Salt  Whole grains | ≥6 g/100 g  ≤0.1 g/100 g  ≥50% | Pasta | Whole grain pasta |
| Meat products | |  |  |  |  |
|  | Sausages, whole | Total fat  Total sugars  Salt | ≤10 g/100 g  ≤3.0 g/100 g  ≤2.0 g/100 g | Hot dog, pork and beef mix | Hot dog, poultry |
|  | Sausages, cold cuts | Total fat  Total sugars  Salt | ≤10 g/100 g  ≤3.0 g/100 g  ≤2.2 g/100 g | Salami | Smoked turkey |
|  | Liver pate | Total fat  Total sugars  Salt  Meat content | ≤10 g/100 g  ≤3.0 g/100 g  ≤1.7 g/100 g  ≥35% | Liver pate (26%) | Liver pate (10%) |
|  | Smoked meat, cold cuts | Total fat  Total sugars  Salt | ≤10 g/100 g  ≤3.0 g/100 g  ≤2.5 g/100 g | Smoked ham | Smoked turkey |
| Vegetable products | |  |  |  |  |
|  | Vegetable products | Total fat  Saturated fats  Total sugars  Salt  Grains,  of which whole grains | ≤10 g/100 g  ≤3.5 g/100 g  ≤3.0 g/100 g  ≤1.0 g/100 g  ≥50%  100% | Soybean sausage | Soybean and wheat protein vegetarian meatballs |
| Fat spreads and blends | |  |  |  |  |
|  | Fat spreads | Total fat  Saturated fats  Salt | ≤80 g/100 g  ≤33 % of total fat  ≤1.1 g/100 g | Butter and oil blend (75%) | Margarine (70%) |
|  | Liquid fat blends | Saturated fats  Salt | ≤20 % of total fat  ≤1.0 g/100 g | Liquid butter and oil blend (80%) | Liquid margarine (79%) |
